# Supplementary material for: Natural variants suppress mutations in hundreds of essential genes
Source: Mol Syst Biol. 2021 May 27;17(5):e10138. doi: 10.15252/msb.202010138 (PMC8156963; doi:10.15252/msb.202010138)
Supplement: Supplementary file 1 — Appendix [file MSB-17-e10138-s010.pdf]

Appendix for

Natural variants suppress mutations in hundreds of essential genes

Leopold Parts, Amandine Batté, Maykel Lopes, Michael W. Yuen, Meredith Laver, Bryan-Joseph San Luis, Jia-Xing Yue, Carles Pons, Elise Eray, Patrick Aloy, Gianni Liti, and Jolanda van Leeuwen

Correspondence to: leopold.parts@sanger.ac.uk (L.P.); jolanda.vanleeuwen@unil.ch (J.v.L.)

Table of Contents

Item	Page
Appendix Figure S1. Fitness values at 26 and 34 °C across strains and replicates	2-3
Appendix Figure S2. Suppression score distributions	4
Appendix Figure S3. Number of suppression events using alternative reference measurements	5

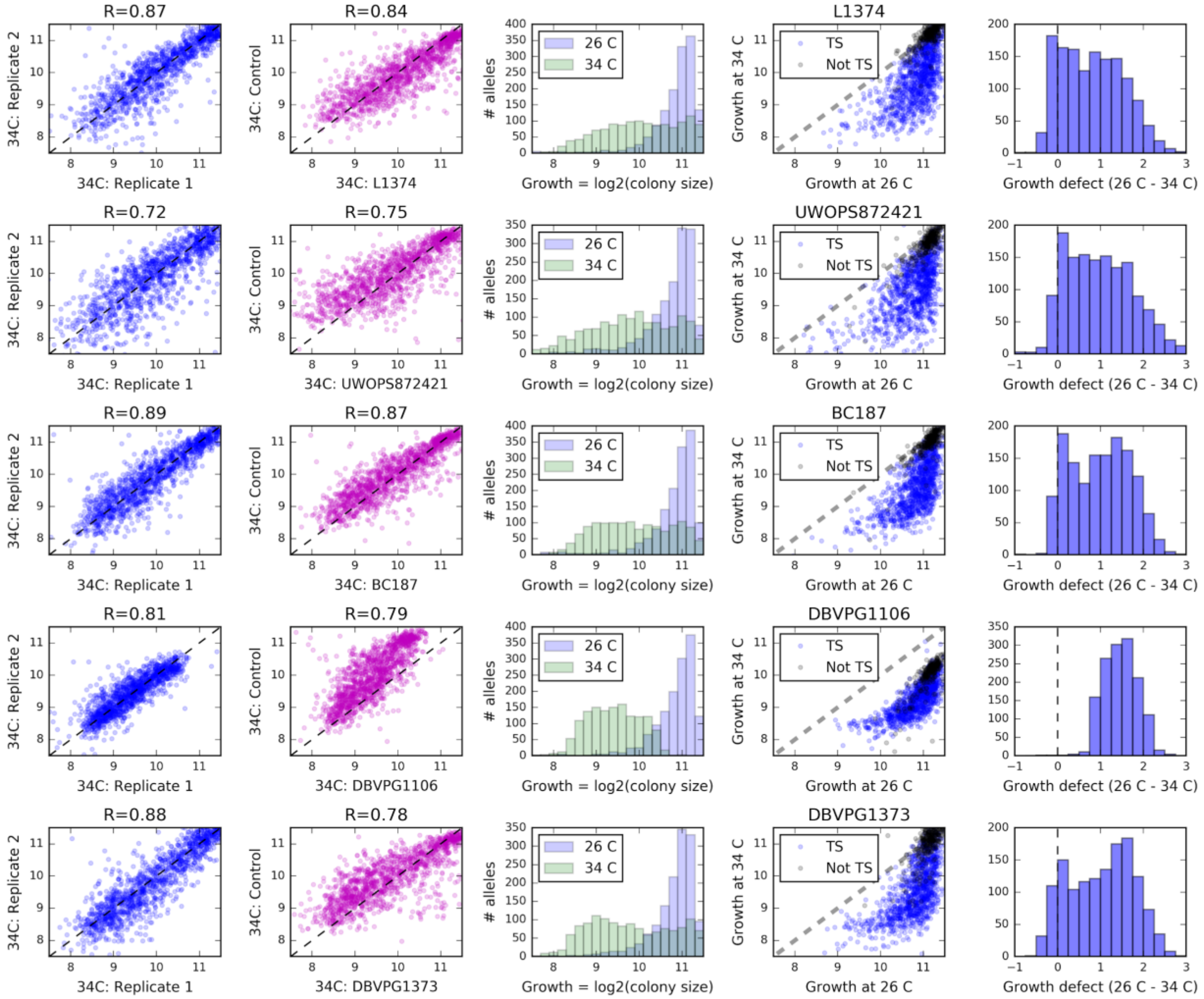

Continued on the next page

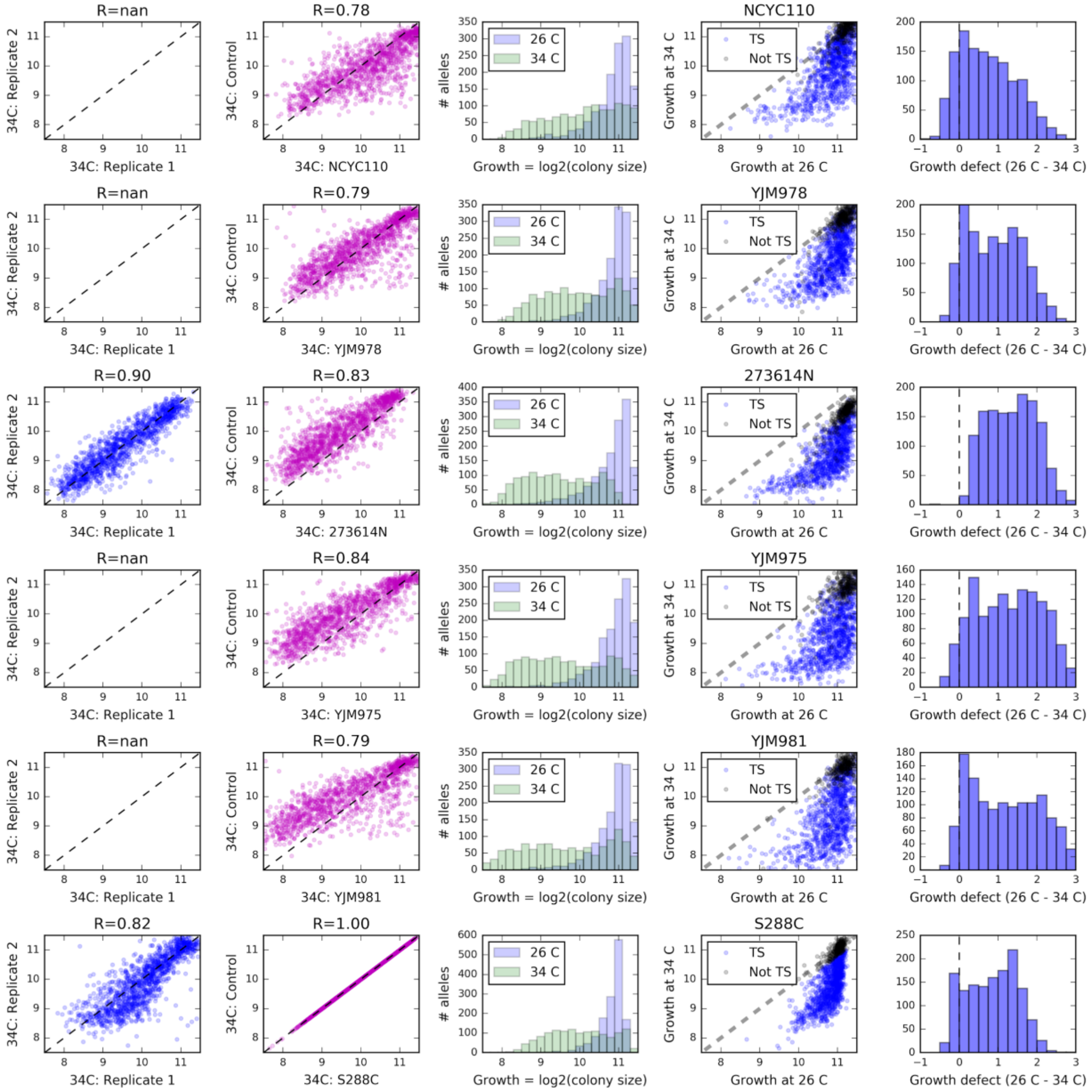

Appendix Figure S1. Fitness values at 26 and 34 °C across strains and replicates. Rows: ten wild strains and the reference (S288C). First column: fitness (log2-scale growth) of TS allele to wild strain cross progeny in biological replicate 1 (x-axis) and biological replicate 2 (y-axis) at 34°C. Dashed line: y=x. Title: Pearson's correlation coefficient. Second column: fitness (log2-scale growth) of TS allele to wild strain cross progeny (x-axis) and to control strain cross progeny (y-axis) at 34°C. Dashed line: y=x. Title: Pearson's correlation coefficient. Third column: frequency (y-axis) of TS allele to wild strain cross progeny growth (x-axis; log2-scaled colony size in pixels) at 26°C (blue) and 34°C (green). Fourth column: growth at 26°C (x-axis) and 34°C (y-axis) of the same progeny as in middle column. Black: TS alleles deemed not temperature sensitive based on the reference cross. Blue: all other TS alleles. Fifth column: frequency (y-axis) of temperature sensitive growth defect (growth at 26°C minus growth at 34°C; x-axis). Dashed line: No temperature sensitive growth defect.

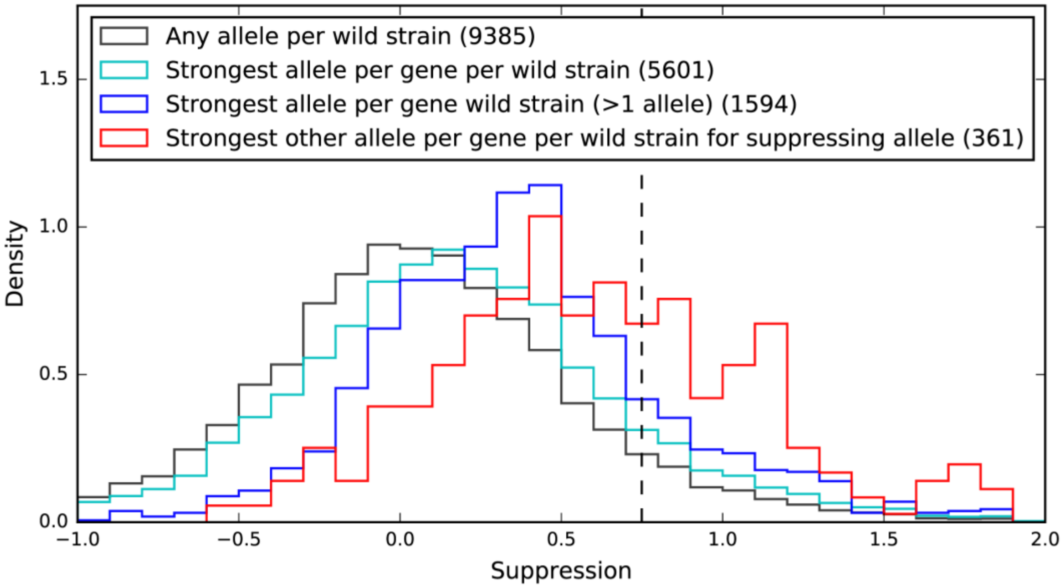

Appendix Figure S2. Suppression score distributions. Density (y-axis) of suppression scores (x-axis) for all TS allele and wild strain combinations (black, N=9,385 measurements), for the strongest suppression effect per gene per wild strain (cyan, N=5,601 measurements), for the strongest suppression effect per gene per wild strain for genes with at least two measured TS alleles (blue, N=1,594 measurements), and for the strongest suppression effect for another TS allele per gene per wild strain given one TS allele had a suppression score above 0.75 (red, N=361 measurements). Dashed line: a suppression score of 0.75, which is used to call suppression in the main text and to filter measurements for the red distribution in this figure.

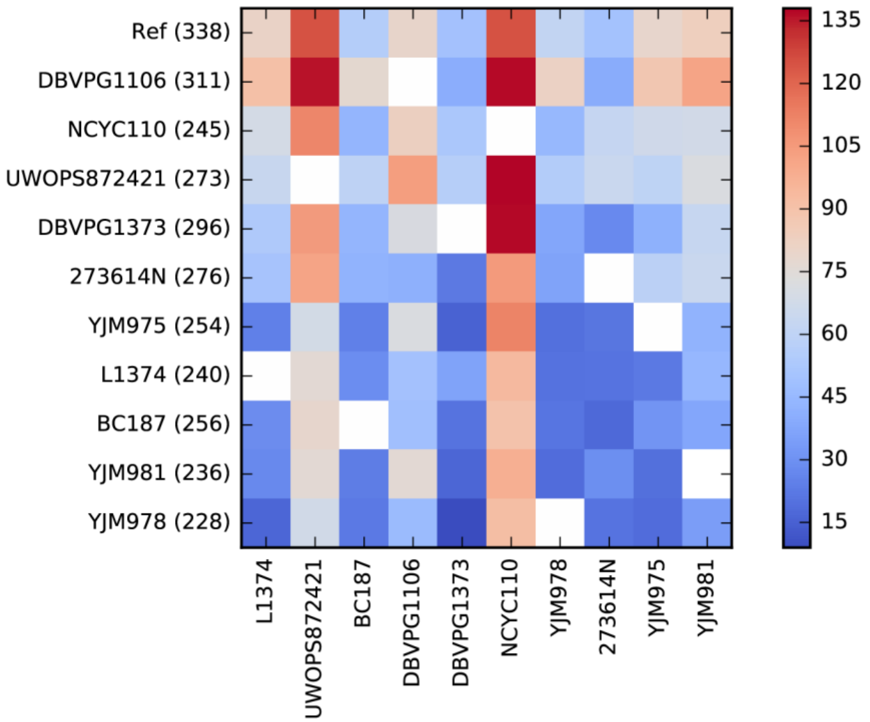

Appendix Figure S3. Number of suppression events using alternative reference measurements. Number of TS alleles (color) suppressed by a wild strain (x-axis) with at least 0.75 log2-scale growth increase at 34°C compared to a given reference strain (y-axis). Number in parentheses: total number of TS alleles with a growth increase of at least 0.75 when the indicated strain is used as a reference. White squares: self-comparisons. Ref: the standard reference strain (S288C).
