# Supplementary material for: Natural variants suppress mutations in hundreds of essential genes
Source: Mol Syst Biol. 2021 May 27;17(5):e10138. doi: 10.15252/msb.202010138 (PMC8156963; doi:10.15252/msb.202010138)
Supplement: Supplementary file 2 — Expanded View Figures PDF [file MSB-17-e10138-s006.pdf]

Expanded View Figures

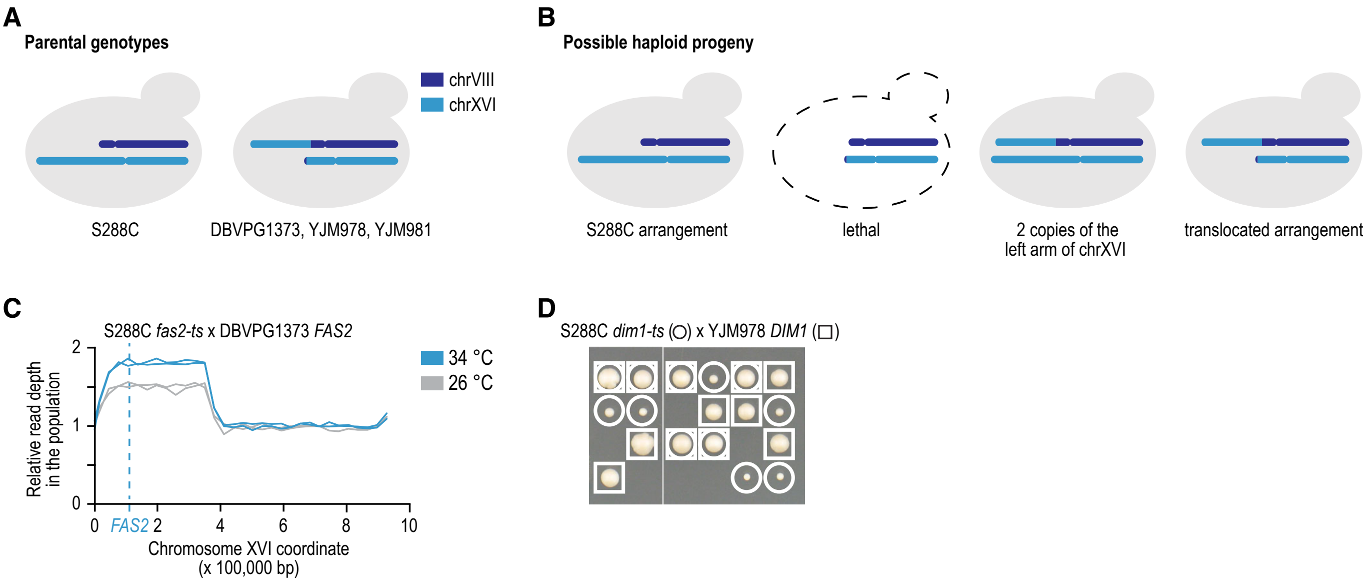

Figure EV1. Suppression by a translocation between chrVIII and chrXVI.

A Three of the strains used in our screens carried a translocation between the promoter regions of *ECM34*, located at the left arm of chrVIII, and *SSU1*, located at the left arm of chrXVI (Pérez-Ortín *et al*, 2002).

B Crossing S228C to one of the strains carrying the translocation will result in 25% spore lethality, due to the absence of a large part of the left arm of chrXVI, which is essential for viability. Of the viable spores, one third will carry the S288C chromosome arrangement, one third will carry the arrangement of the translocated strain, and one third will carry the S288C version of chrXVI, combined with the rearranged chromosome that carries the chrVIII centromere. This last combination of chromosomes will lead to the presence of two copies of a large part of the left arm of chrXVI.

C, D A second copy of part of the left arm of chrXVI can suppress the TS phenotype of TS alleles located on this part of chrXVI. *FAS2* (*YPL231W*) and *DIM1* (*YPL266W*) are located on the translocated left arm of chrXVI. (C) Population sequencing read depth of haploid progeny carrying a *fas2-ts* allele, isolated from a cross between a S228C *fas2-ts* mutant and a DBVPG1373 wild-type strain, at either 26 or 34°C. Part of the left arm of chrXVI is present at increased copy number in the population at 26°C, due to the lethality of spores lacking the chromosome arm. At 34°C, selection occurs for cells carrying two copies of this chromosomal fragment, because in addition to carrying the TS allele, they will also carry a wild-type copy of *FAS2* that is inherited from DBVPG1373. (D) Tetrad dissection of a cross between a S288C *dim1-ts* mutant and a YJM978 wild-type strain. Cells carrying both a *dim1-ts* and a *DIM1* wild-type allele have increased fitness compared to mutants carrying only a *dim1-ts* allele.

© 2021 The Authors

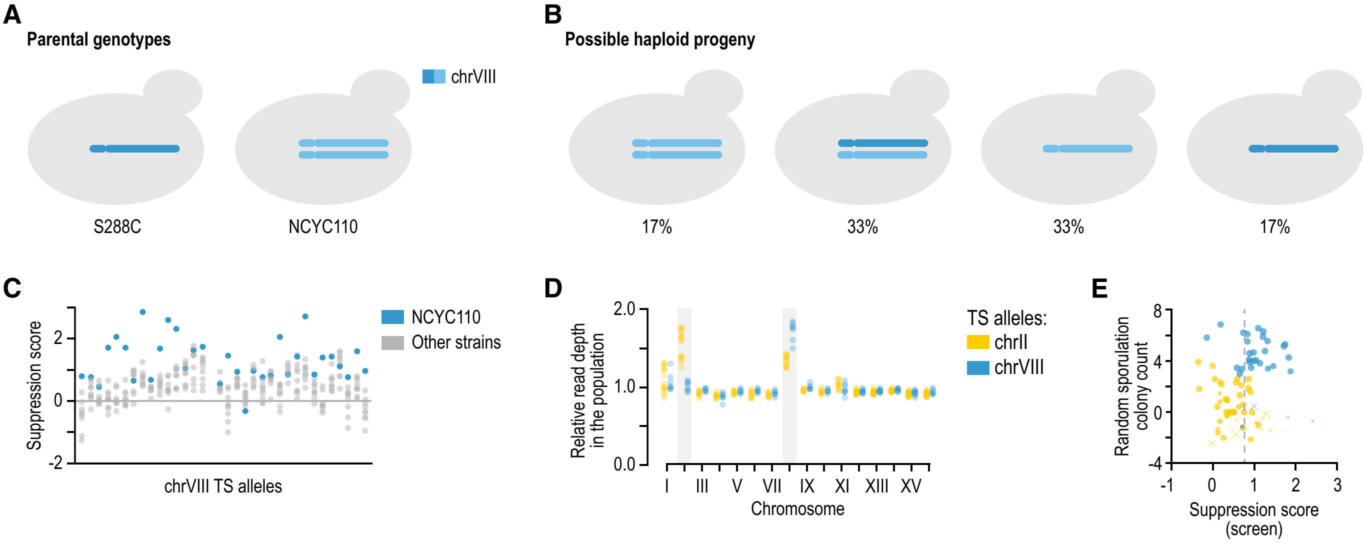

Figure EV2. Aneuploidies and suppression validation.

A, B The NCYC110 strain that was used in our screen carried aneuploidies of chromosomes II and VIII. Chromosome VIII is highlighted as an example. Crossing S228C to NCYC110 will result in 50% of the progeny carrying one copy of chrVIII, and 50% of the progeny carrying two copies of chrVIII. Of the progeny carrying one copy of chrVIII, one third will carry the S288C chromosome and two thirds the NCYC110 chromosome. Of the progeny carrying two copies of chrVIII, one third will carry two NCYC110 chromosomes, and two thirds will carry one S288C and one NCYC110 version of chrVIII. We note that recombination can occur between the S288C and NCYC110 chromosomes.

C, D A second copy of a chromosome can suppress the TS phenotype of TS alleles located on this chromosome. (C) For each TS allele located on chrVIII, the suppression scores are plotted for haploid progeny of a cross to NCYC110 (blue) or other wild isolates (grey). (D) Shown are the relative sequencing read depth per chromosome of populations of haploid progeny isolated from a cross of NCYC110 to TS alleles located on either chrII (yellow) or chrVIII (blue). Cells carrying a TS allele on chrVIII have substantially higher coverage of this chromosome compared to query genes located on chrII. Note that aneuploidy of chromosome II is lost in the absence of positive selection, suggesting that it is detrimental in the population.

E Screen and individual colony scores are concordant. Screen suppression score (*x*-axis; $\log_2$-scale growth ratio between wild strain and reference crosses at 34°C; Methods) and individual colony count difference (*y*-axis; $\log_2$-scale colony count ratio in the wild strain cross between 26 and 34°C minus $\log_2$-scale colony count ratio in the reference cross between 26 and 34°C). Marker size: individual colony count at permissive temperature (smaller markers for fewer colonies); marker type: "x" for TS strains with low temperature sensitivity in the control cross (colony count ratio between 26 and 34°C below 2); "o" for the rest; marker colour: blue for crosses with visually confirmed suppression; yellow for unconfirmed. Note that visual confirmations also include cases of increased colony size, not solely increased colony number (Dataset EV3). Dashed grey line: the cut-off used for calling suppression in the screen.

 © 2021 The Authors

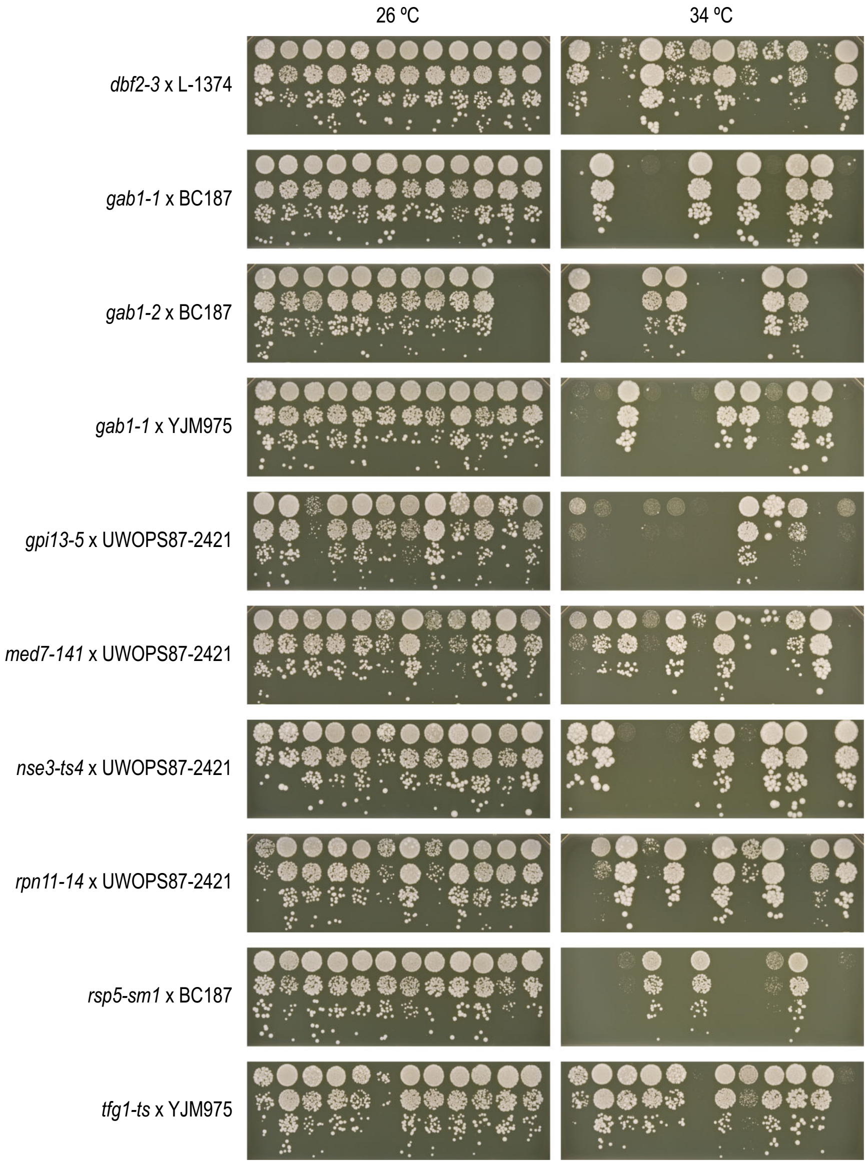

Figure EV3. Estimating the number of strong modifiers.

TS alleles were crossed to the wild strain in which they were suppressed, and the resulting hybrid strains were dissected at 26°C. The first 12 spores carrying the TS allele were selected from the dissection plate and were grown overnight in liquid media. Cultures were diluted to an optical density at 600 nm of 0.1 and a series of tenfold dilutions was spotted on agar plates and incubated for 2 days at 26°C or 34°C. The number of strong modifiers was estimated by determining the fraction of the spores that grew well at 34°C.

Figure EV4. Suppressor identification.

To validate the suppressor candidates, we replaced the suppressor gene reference alleles with their wild version in the reference strain background and tested for suppression of the corresponding TS allele. Cultures of the indicated strains were grown until saturation, and a series of tenfold dilutions was spotted on YPD agar plates and incubated at the indicated temperatures. See Fig 5A for the suppression of *nse3-ts4* by *NSE1-UWOPS872421*.

▶

© 2021 The Authors

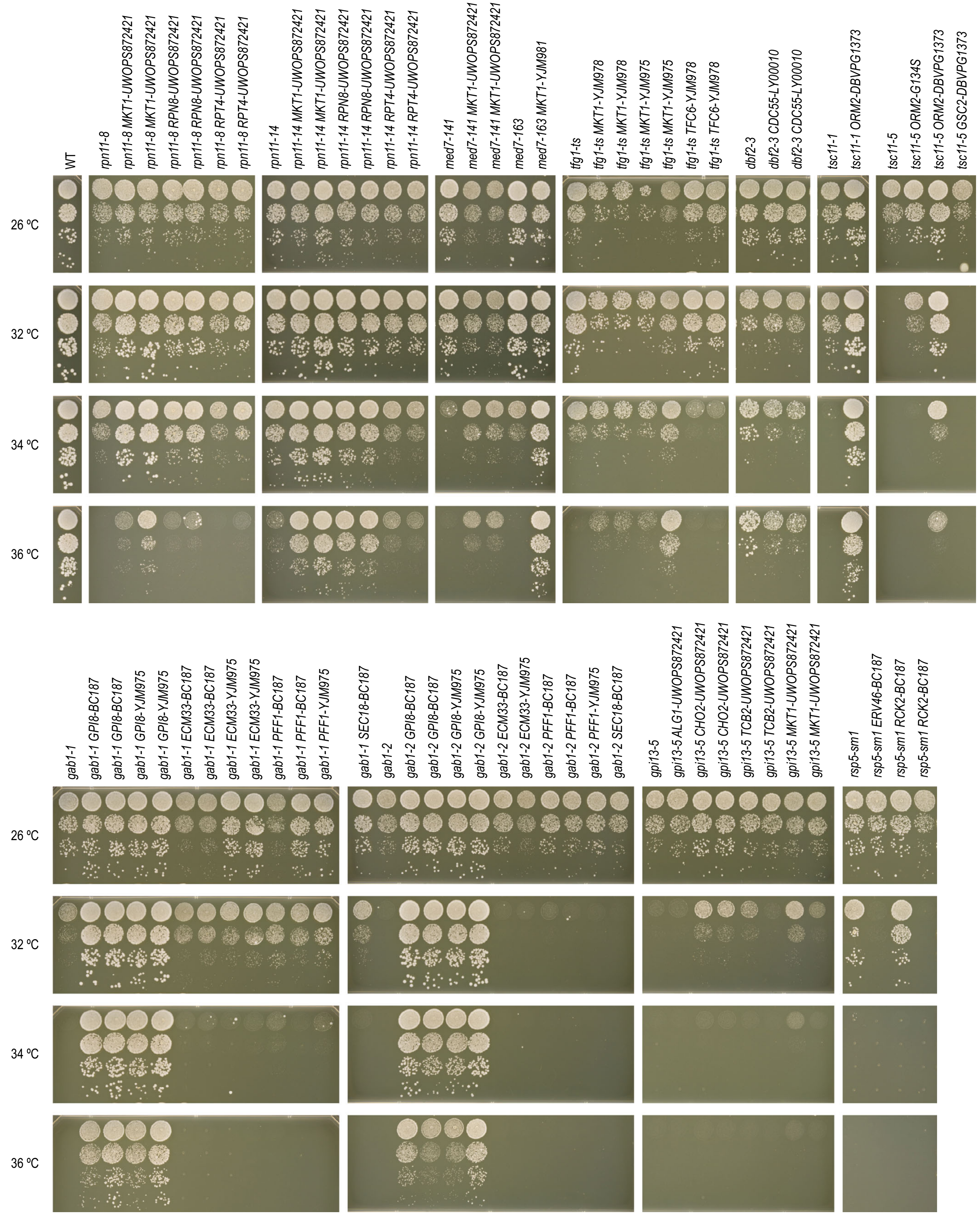

Figure EV4.

 © 2021 The Authors

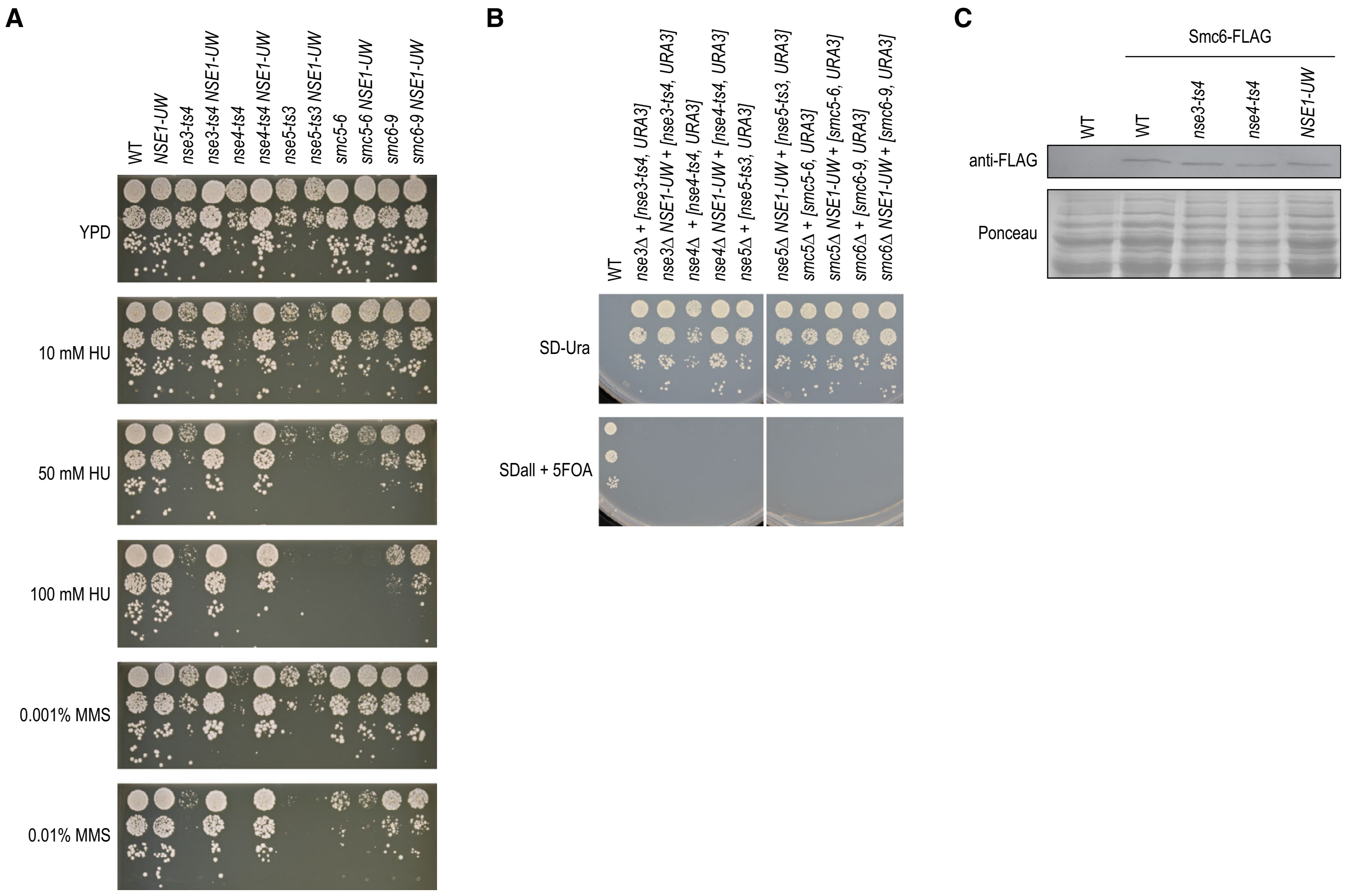

Figure EV5. The *NSE1* allele of UWOPS87-2421 can suppress *NSE3* and *NSE4* TS mutants, but not deletion mutants.

A Suppression of the DNA damage sensitivity of *nse3-ts4* and *nse4-ts4* TS mutants by the *NSE1* allele of UWOPS87-2421. Cultures of the indicated strains were diluted to an optical density at 600 nm of 0.1 and a series of tenfold dilutions was spotted on agar plates and incubated for 2–3 days at 30°C. UW = UWOPS87-2421, WT = wild type.

B The *NSE1-UW* allele cannot suppress the lethality associated with deletion alleles of genes encoding SMC5/6 complex members. Spot dilutions were performed as described in (A).

C Smc6-FLAG levels are not affected by mutations in *NSE1*, *NSE3*, or *NSE4*. Western blot analysis of Smc6-FLAG in the indicated strains. Ponceau staining was used as a loading control.
